# The Breath, and Diseases Which Give It a Fetid Odor

**Published:** 1874-12

**Authors:** 


*The Breath and the Diseases which give it a Fetid Odor, with directions for treatment.* By Joseph W. Howe, M. D. New York: D. Appleton & Co., 1874. Buffalo: Martin Taylor.

An offensive breath is a vexation which the physician is frequently called upon to relieve, and unless the cause is apparent in decayed teeth, some digestive disorder or other easily detected malady, the medical practitioner is very liable to fail or to give at best only temporary relief. Dr. Howe has given considerable attention to this subject, and has embodied his ideas and discoveries in a book of a little over one hundred pages.

The book is composed of seven chapters, which discuss the physiology of repair, decay and respiration, fetid ordors from emotion, etc, fetid odors from dispepsia, from bad teeth and ulcers of the mouth, from catarrhal disorders, and from mineral poisons.

In chapter second he considers fetid ordors from emotion and shows by report of cases etc, that excessive or unusual mental emotion may produce a fetid breath. He advises the prevention of all nervous excitement, the employment of nervous tonics, etc. The varied causes and conditions of fetid breath are discussed at some length, and from a cursory examination we are inclined to the opinion that a careful examination of the work by physicians will enable them to more frequently relieve their distressed patients.

